# An Atypical Presentation of Sarcoidosis

**DOI:** 10.7759/cureus.67406

**Published:** 2024-08-21

**Authors:** Chelsea J Kubinec, Zaeem A Siddiqi, Lauren Bolster, Christopher Fung, Aldo J Montano-Loza, Safwat Girgis, Jennifer Ringrose

**Affiliations:** 1 Medicine, University of Alberta, Edmonton, CAN; 2 Radiology and Diagnostic Imaging, University of Alberta, Edmonton, CAN; 3 Laboratory Medicine and Pathology, University of Alberta, Edmonton, CAN

**Keywords:** splenomegaly, case report, calcium deposition, hypercalcemia, sarcoidosis

## Abstract

Sarcoidosis is a multisystem disease characterized by non-caseating granulomatous organ infiltration. We describe an atypical presentation of sarcoidosis in a 43-year-old male presenting with fatigue and shortness of breath. He had a preceding history of recurrent venous thromboembolism (VTE), hemolytic anemia, cirrhosis, peripheral neuropathies, and calcium deposition, which pre-dated hypercalcemia; he was later diagnosed with IgA nephropathy. Clinicians should consider sarcoidosis in patients with findings of multisystem disease even without hilar lymphadenopathy or hypercalcemia.

## Introduction

Sarcoidosis is characterized by non-caseating granulomatous infiltration of organs [1]. It can affect the lungs, heart, liver, kidneys, and nervous system [1]. The incidence and prevalence of sarcoidosis varies globally [2]. In Canada, the prevalence of sarcoidosis was 66 cases per 100,000 in 1996 and 143 cases per 100,000 in 2015 [3]. The etiology of sarcoidosis is unknown. Diagnosis is based on clinical presentation in keeping with sarcoidosis, non-caseating granulomas identified on histology, and exclusion of other diseases known to cause granulomatous infiltration [4]. Histological confirmation is unnecessary in specific cases where clinical suspicion is high (Löfgren’s syndrome, lupus pernio, Heerfordt’s syndrome) [4]. We describe an atypical case of sarcoidosis in a 43-year-old male.

## Case presentation

The patient initially presented to the hospital with fatigue and shortness of breath. He was found to have severe anemia (hemoglobin 49 g/L), positive hemolytic markers, and an enlarged spleen (19 cm) and required a total of five units of packed red blood cells (laboratory investigations are shown in Table 1). His medical history is significant for hypertension, recurrent venous thromboembolism (VTE), hemolytic anemia, splenomegaly, tophaceous gout, liver transplantation for cirrhosis, nephrolithiasis, gallbladder calcinosis, hypercalcemia, and micronutrient deficiencies. He has also been diagnosed with IgA nephropathy with ongoing albuminuria.

**Table 1:** Laboratory results. N/A: not applicable, 1 GPL: 1 microgram of IgG antibody.

Test	Result	Normal Range
Initial presentation		
Hemoglobin	49 g/L	135-175 g/L
Mean corpuscular volume (MCV)	119 fL	80-100 fL
Platelets	136 x 10^9^	140-150 x 10^9^
While blood cells (WBCs)	1.9 x 10^9^	4.0-11.0 x 10^9^
Fibrinogen	2.4 g/L	1.9-4.1 g/L
Haptoglobin	<0.10 g/L	0.30-2.00 g/L
Bilirubin, total	58 µmol/L	<20 µmol/L
Alanine transaminase (ALT)	50 U/L	<50 U/L
Alkaline phosphatase	69	30-130 U/L
Aspartate transaminase (AST)	67 U/L	<40 U/L
Lactate dehydrogenase	295 U/L	100-225 U/L
Activated partial thromboplastin clotting time (APTT)	28 seconds	27-38 seconds
International normalized ratio (INR)	1.3	0.8-1.2
Serum protein electrophoresis pattern	Normal	N/A
Urine protein electrophoresis	Normal pattern, small amount of albumin present	N/A
Albumin	35 g/L	35-50 g/L
Ferritin	476 µg/L	12-300 µg/L
Vitamin B12	123 pmol/L	>150 pmol/L
Folate	1.2 nmol/L	>9.9 nmol/L
Erythrocyte sedimentation rate (ESR)	99 mm/h	0-15 mm/h
Direct antiglobulin test	Negative	N/A
Donath Landsteiner Ab	Negative	N/A
Paroxysmal nocturnal panel	Normal	N/A
Osmotic fragility (eosin 5-maleimide median fluorescence)	105%	90%-110%
Peripheral smear	Severe macrocytic anemia with oval macrocytes, teardrop poikilocytes and hypersegmented neutrophils	N/A
Cold agglutinin; titer	Negative	N/A
Bone marrow biopsy	Hypercellular marrow with megaloblastic erythropoiesis and granulopoiesis	N/A
Flow cytometry	CD5/CD10-negative monoclonal kappa B-cells detected	N/A
Second presentation		
Hemoglobin	135 g/L	135-175 g/L
Folate	>40 nmol/L	>9.9 nmol/L
Vitamin B12	183 pmol/L	>150 pmol/L
Selenium	0.81 µmol/L	1.29-2.60 µmol/L
Calcium	1.93 mmol/L	2.10-2.60 mmol/L
Albumin	26 g/L	35-50 g/L
Ionized calcium	1.01 mmol/L	1.09-1.25 mmol/L
Magnesium	0.46 mmol/L	0.70-1.00 mmol/L
Tissue transglutaminase Ab IgA	<1/0 U/mL	<7.0 U/mL
Vitamin C	16 µmol/L	35-95 µmol/L
Zinc	6.5 µmol/L	8-20.0 µmol/L
Hypercalcemia investigations		
Calcium, Urine	16.4 mm/V	2-7.5 mmol/V
Calcium	2.9-3.12 mmol/L	2.10-2.60 mmol/L
Ionized calcium	1.43 mmol/L	1.09-1.25 mmol/L
Albumin	30 g/L	35-50 g/L
Angiotensin-converting enzyme	125-194 U/L	13-57 U/L
Parathyroid hormone (PTH)	0.7 pmol/L	1.4-6.8 pmol/L
Phosphate	1.00 mmol/L	0.8-1.45 mmol/L
1,25-dihydroxyvitamin D	48 pmol/L	42-168 pmol/L
25-hydroxyvitamin D	48 nmol/L	80-200 nmol/L optimum level
Cerebrospinal fluid albumin	28 g/L	35-50 g/L
Cerebrospinal fluid protein	0.45 g/L	0.15-0.45 g/L
Cerebrospinal fluid pathology	Negative for malignancy	N/A
General		
Human immunodeficiency virus (HIV) 1+2	Non-reactive	N/A
Immunoglobulin G	16.15 g/L	6.94-16.18 g/L
Immunoglobulin A	11.00 g/L	0.70-4.00 g/L
Immunoglobulin M	1.51 g/L	0.60-3.00 g/L
Anti-neutrophil cytoplasmic antibody (ANCA)	Negative	N/A
Extractable nuclear antibody (ENA)	Negative	N/A
Janus kinase 2 (JAK2) V617F bone marrow	Negative	N/A
Lupus anticoagulant	Negative	N/A
Factor V Leiden	Negative	N/A
Anti-cardiolipin antibody	<15 GPL units	<15 GPL units
Anti-beta 2 glycoprotein	<1.4 U/mL	<19.9 U/mL

During the initial presentation, his vitamin B12 and folate levels were low, and replacement was initiated. The direct antibody test (DAT) was negative. The peripheral blood smear demonstrated red cell agglutination. Other markers including cold agglutin titers, paroxysmal nocturnal hemoglobinuria (PNH), Donath-Landsteiner antibody, and osmotic fragility eosin 5-maleimide median fluorescence (EMA) assay were negative. Bone marrow biopsy showed a hypercellular marrow with megaloblastic erythropoiesis and granulopoiesis. Flow cytometry on the bone marrow biopsy was suggestive of CD5/CD10 negative B-cell lymphoproliferative disorder. Given the flow cytometry result and the splenomegaly, there was concern for marginal cell lymphoma. Ultimately, the patient underwent a laparoscopic splenectomy. Splenic pathology showed extravascular hemolysis and did not demonstrate evidence of lymphoproliferative disease or granulomatous infiltration. He was initially started on empiric corticosteroids for hemolytic anemia which were tapered post splenectomy and hemoglobin remained stable. Serum protein electrophoresis repeatedly demonstrated no monoclonal protein. Urine protein electrophoresis showed no evidence of monoclonal protein, although it revealed proteinuria consistent with glomerular/tubular damage. Immunoglobulin free light chains consistently showed a normal kappa/lambda ratio.

Two weeks after his presentation with hemolytic anemia, he presented to the emergency department with paresthesias in all extremities and leg weakness. He was found to have bilateral cranial nerve six palsies. Imaging (CT head, MR brain, and MR cervical/thoracic spine) did not identify a lesion. Laboratory investigations revealed multiple nutritional deficiencies, including corrected folate deficiency, corrected vitamin B12 deficiency, selenium, calcium, magnesium, vitamin C, and zinc deficiencies (Table 1). Investigations for malabsorption were negative (anti-tissue transglutaminase negative, upper endoscopy showed gastritis, and lower endoscopy showed a 4-mm polyp). Electromyography and nerve conduction studies showed a severe length-dependent sensorimotor axonal peripheral neuropathy. These findings were suspected to be secondary to his micronutrient deficiencies, etiology unknown.

Due to liver cirrhosis and subsequent liver failure, the patient underwent liver biopsies (both trans-jugular and ultrasound-guided) with pathology significant for steatohepatitis. The patient later underwent liver transplantation. His explanted liver pathology showed features consistent with steatohepatitis without evidence of granulomas. 

**Figure 1:**
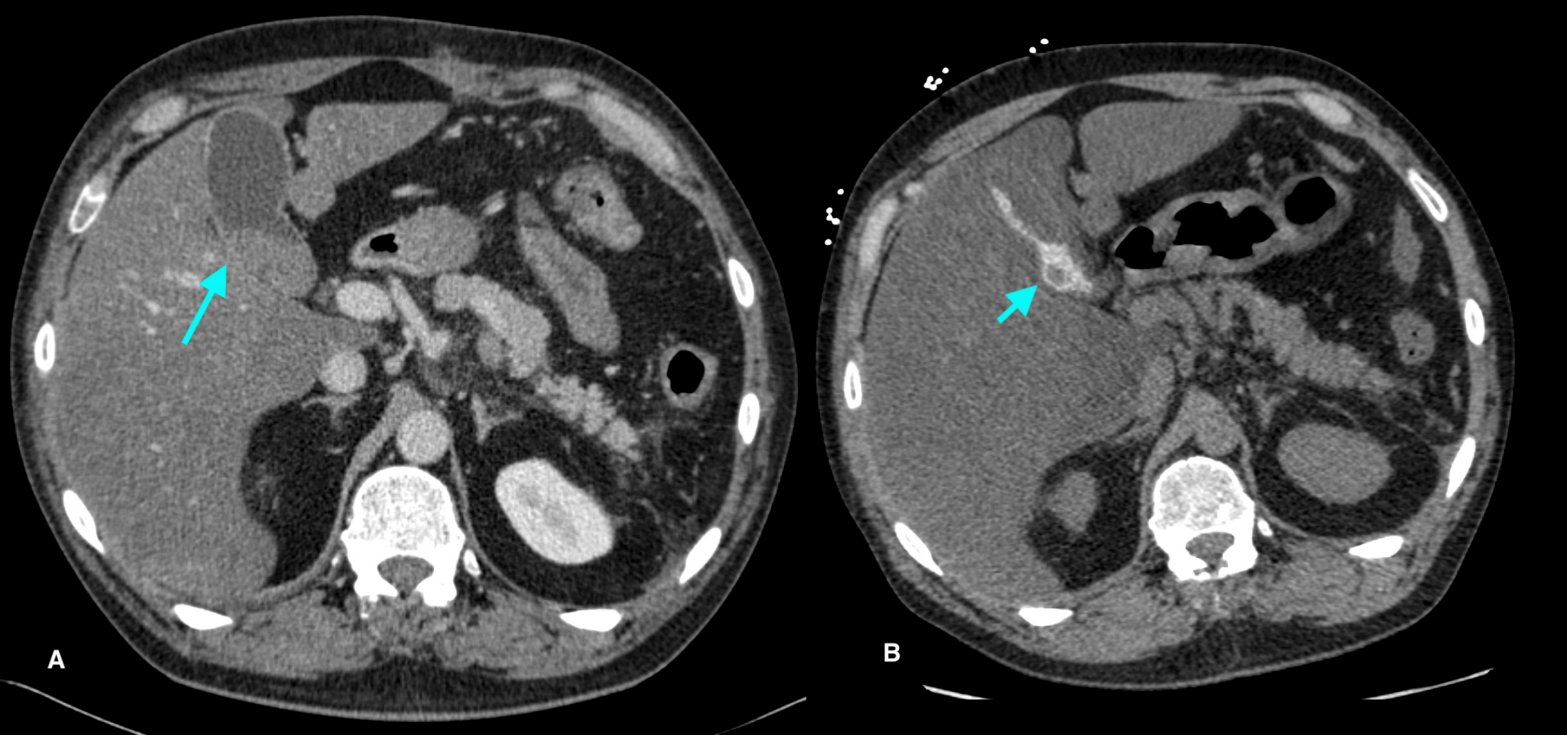
Computed tomography. A. Portal venous phase CT shows layering sludge and debris in the gallbladder at initial presentation in 2017 (A, long arrow). B. Subsequent un-enhanced CT performed one year later (2018) shows a dramatic change with lobulated, layering calcification along the gallbladder wall (B, short arrow).

**Figure 2:**
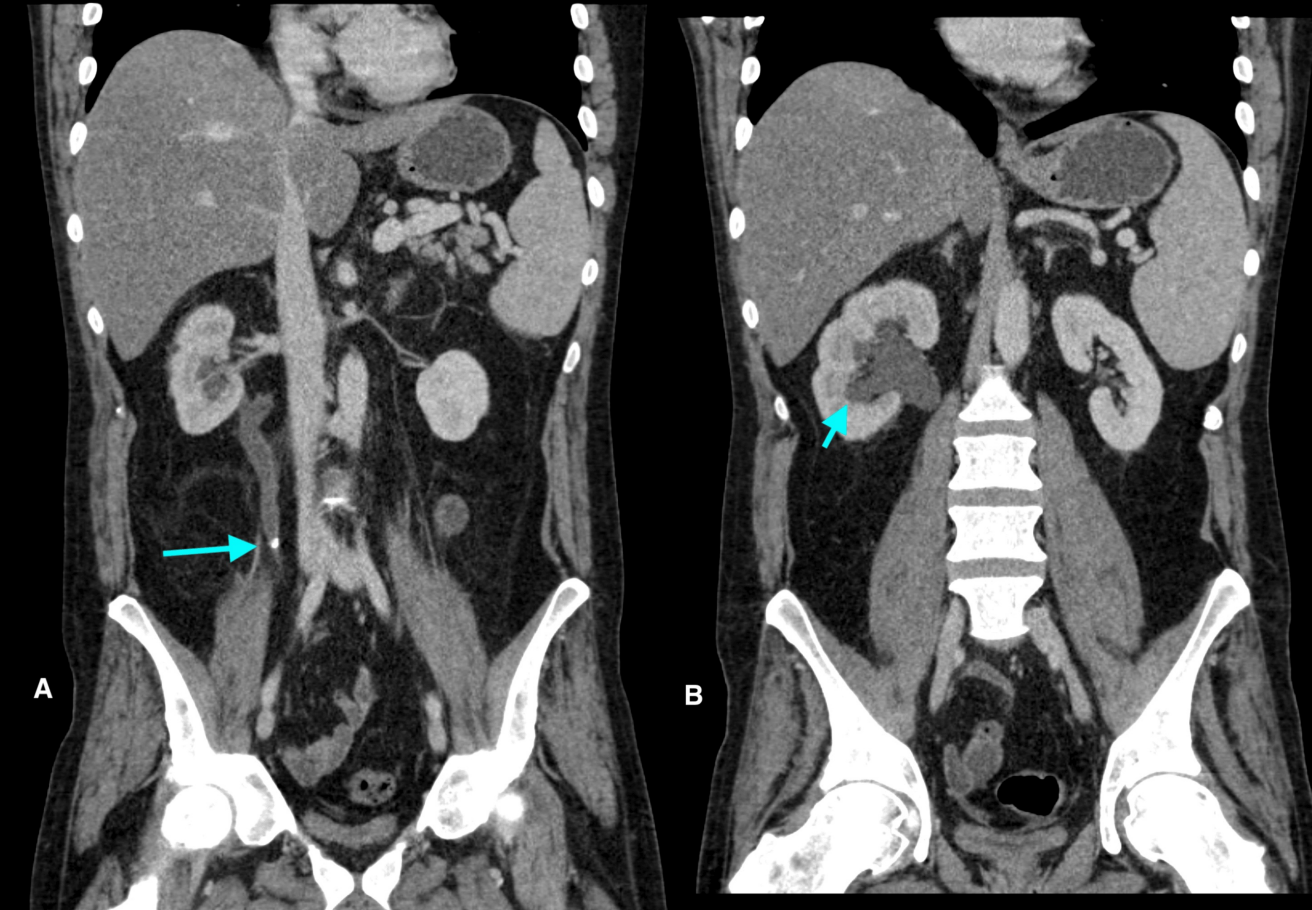
Computed tomography. Initial CT at presentation in 2017 (left) shows an intraureteric right renal calculus (A, long arrow) with upstream moderate hydroureter and hydronephrosis (B, short arrow).

**Figure 3:**
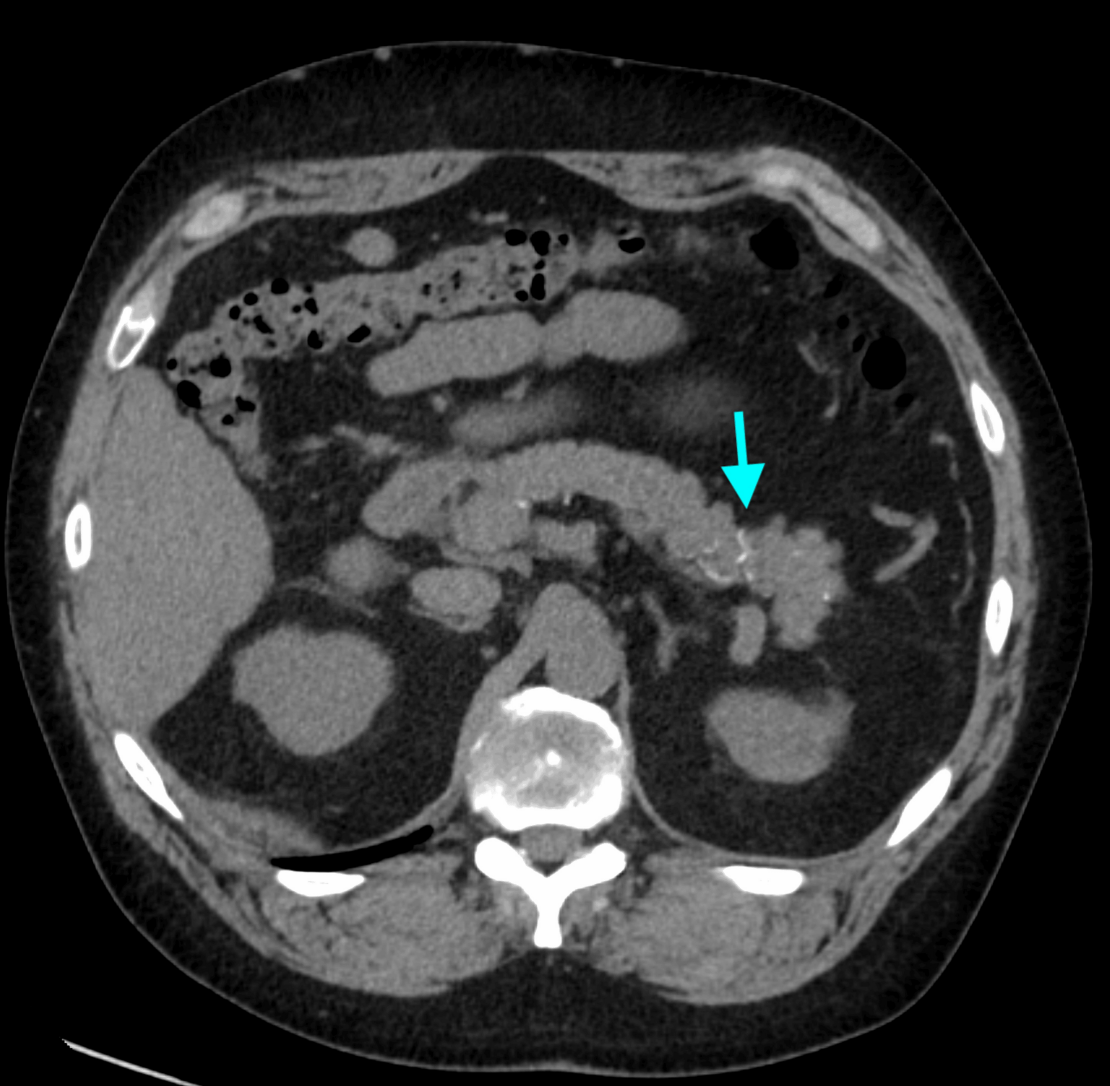
Repeat CT in 2021, four years after initial presentation, shows development of unusual small vessel calcification within the pancreatic tail (arrow).

**Figure 4:**
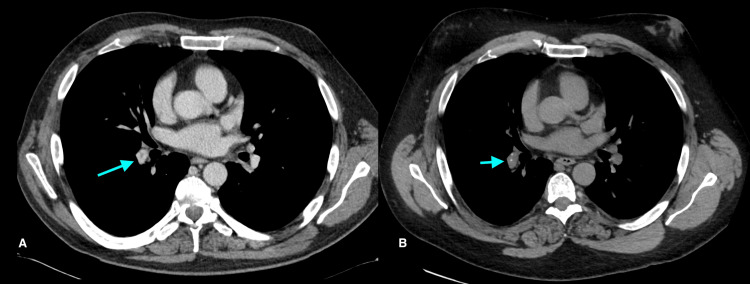
Chronic pulmonary embolus on initial CT (long arrow). B. Calcification of the chronic thrombus four years later (short arrow). A. Chronic pulmonary embolus in the right interlobar pulmonary artery on initial presenting CT in 2017 (left, long arrow). B. Calcification of the chronic thrombus developed in on follow-up CT four years later (right, short arrow).

**Figure 5:**
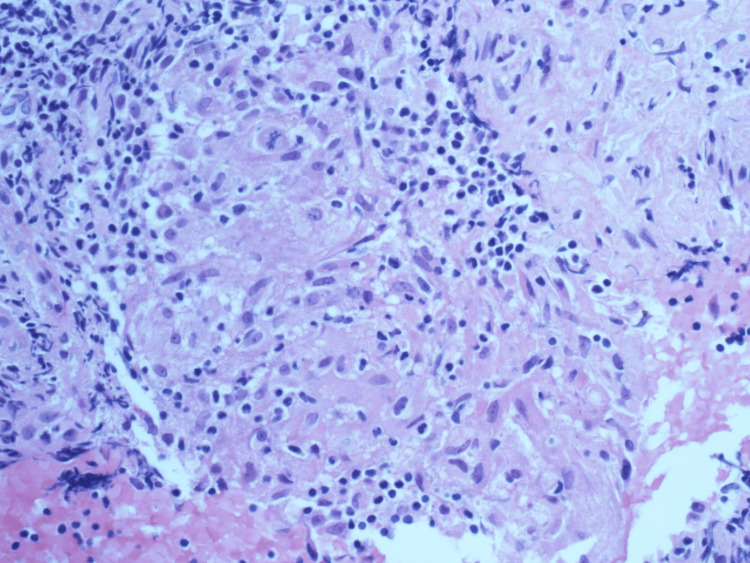
Routine hematoxylin and eosin (H&E)-stained section of endobronchial ultrasound-guided biopsy of 11R hilar node. High-power (40×) view showing non-caseating granuloma with associated benign lymphoid cells and no evident necrosis.

Prior to liver transplantation, the patient had symptomatic hypercalcemia, prompting the consideration of sarcoidosis. His angiotensin-converting enzyme (ACE) level was elevated. He had significant calcification of his gallbladder (porcelain gallbladder) on a CT scan (Figure 1), which was new compared to a CT one year prior. The gallbladder was electively removed given the risk of malignancy. Pathology demonstrated findings in keeping with chronic calculous cholecystitis. He also had a history of hypercalciuria and renal/ureteric stones (Figure 2). He developed small vessel calcification within the pancreatic tail and calcification of a chronic pulmonary thrombus (Figures 3, 4). Except for subtle mediastinal lymphadenopathy, he had no other radiographic findings of sarcoidosis. Pulmonary function testing was normal. He underwent a biopsy of the mediastinal lymphadenopathy which demonstrated non-necrotizing granulomas on pathology, confirming the diagnosis of sarcoidosis (Figure 5). Retrospectively, review of the splenectomy pathological specimens revealed no evidence of granulomas.

This patient’s HIV and hepatitis status have repeatedly tested negative. His cerebrospinal fluid (CSF) sampled in December 2017 showed normal glucose and protein and negative viral and bacterial cultures. He underwent extensive immunological testing, which all resulted negative or within normal range, including markers of vasculitis. When measured, erythrocyte sedimentation rate (ESR) and CRP were appropriately elevated during hospitalizations but normalized as an outpatient. Throughout his course, quantitative immunoglobulin testing would occasionally indicate transient low levels of IgM and IgG; however, none of these immunoglobulin levels were persistently low. 

**Figure 6:**
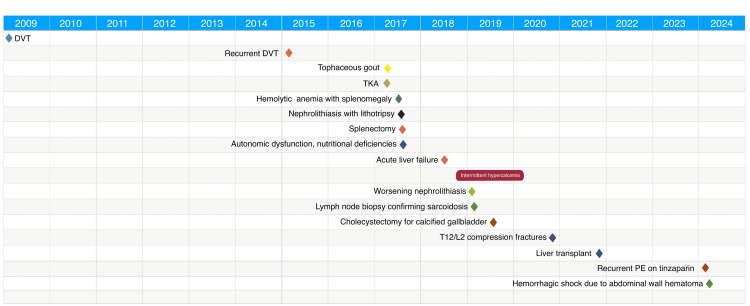
Time course of case presentation. DVT: deep vein thrombosis, TKA: total knee arthroplasty, T: thoracic, L: lumbar, PE: pulmonary embolism.

Thrombophilia testing was performed given his extensive thrombosis history, which showed antithrombin deficiency. Previous testing for antiphospholipid antibody syndrome was negative. He had a negative JAK2 mutation (B617F). Given that there were no clear strong provoking factors for VTE (with recent recurrence on daily therapeutic tinzaparin), he was recently sent for repeat thrombophilia and hereditary thrombophilia workup (anticardiolipin antibody, anti-beta 2 glycoprotein, and factor V Leiden were negative). His course was recently complicated by an abdominal wall hematoma and associated shock, but he is now improving, with graft function and serum calcium stable on tacrolimus, mycophenolate mofetil, and low-dose prednisone. Time course of his case is shown in Figure 6.

## Discussion

Through a retrospective review of this patient’s case, the history of recurrent VTE, hemolytic anemia, calcium deposition, neuropathies, and IgA nephropathy may be related to his eventual diagnosis of sarcoidosis. There is an association between sarcoidosis and VTE, likely related to chronic inflammation [5]. A retrospective cohort study found that sarcoidosis patients were more likely to develop VTE compared to those of the general population [5]. An association exists between the site of sarcoidosis infiltration and the site of thrombosis [6]. Interestingly, our patient developed portal venous thrombosis one year prior to his diagnosis of sarcoidosis.

The etiology of his liver cirrhosis is thought to be multifactorial with possible sarcoidosis involvement. Three liver biopsies, along with the explanted liver pathology, were never able to confirm sarcoid involvement. Concurrent diagnoses of sarcoidosis and hemolytic anemia are rare, although they have been reported in the literature [7]. There appears to be some overlap in the complex pathophysiology underlying both sarcoidosis and autoimmune hemolytic anemia [7]. In our presented case, the patient had a negative DAT and no evidence of spherocytes, despite a low haptoglobin (<0.10 g/L), reticulocytosis (7.6%; 97.3 x 10^9^/L), elevated lactate dehydrogenase (LDH) (344 U/L), and bilirubin (total bilirubin 106 µmol/L). While rare, DAT-negative autoimmune hemolytic anemia is possible, although it may be reflective of inherent limitations of the employed assay. His hemolytic anemia resolved post-splenectomy. 

Neurological involvement in cases of systemic sarcoidosis has a reported incidence between 3.5% and 7.2% [8]. Neurologic involvement of sarcoidosis can present as cranial neuropathies, peripheral neuropathies, pituitary/hypothalamic involvement, brain involvement through pachymeningitis, leptomeningitis, and/or vasculitis [8]. Our patient had transient bilateral cranial nerve six palsies which resolved with treatment of nutritional deficiencies. His lower limb peripheral neuropathy has improved but persists. The etiology of our patient’s peripheral neuropathy is thought to be multifactorial, including vitamin deficiencies, prior alcohol use, gout, and immunosuppressive medications. A nerve biopsy was not performed as this procedure would not change management. 

IgA nephropathy is a common cause of glomerular disease and is characterized by the deposition of IgA antibodies in the glomerular mesangium [9]. Its prevalence is likely underestimated given the requirement of renal biopsy for diagnosis [9]. Like sarcoidosis, it is considered a systemic autoimmune disease [9]. Renal sarcoidosis is an extrapulmonary complication of sarcoidosis. In a small epidemiological study of renal sarcoidosis, Löffler et al. (2014) found that IgA glomerulonephritis was present in 26% of study participants with histologically proven renal sarcoidosis [10]. Non-granulomatous tubulointerstitial nephritis was found in 44% of participants [10]. Our patient was diagnosed with biopsy-proven IgA nephropathy. The kidney biopsy also documented global and segmental glomerulosclerosis, mild interstitial fibrosis and tubular atrophy, and moderate arteriosclerosis. There was no granulomatous infiltration identified.

One study reports hypercalcemia in 6% of sarcoidosis patients [11]. Hypercalciuria is present in 20%-40% of affected patients [12]. Consequently, nephrolithiasis is more common in patients with sarcoidosis, affecting 10%-14% of these patients over their disease course [12]. Our patient had documented hypercalciuria, nephrolithiasis, and ureteric calculus requiring lithotripsy prior to the development of overt hypercalcemia. He had significant gallbladder calcification, which progressed over the course of one year (Figure 1). Furthermore, he had small vessel calcification within the pancreatic tail and calcification of a chronic pulmonary thrombus (Figures 3, 4).

The recurrent VTE, hemolytic anemia, calcium deposition, liver cirrhosis, and cranial and peripheral nerve involvement, and IgA nephropathy may be separate disease processes rather than directly associated with sarcoidosis. However, sarcoidosis may be the shared pathophysiology connecting these entities in this young patient.

## Conclusions

In summary, sarcoidosis is a multisystem disease characterized by non-necrotizing granulomatous infiltration of various organ systems. We describe an atypical case of sarcoidosis with a preceding medical history significant for recurrent VTE, hemolytic anemia, liver cirrhosis, peripheral neuropathies, chronic kidney disease secondary to IgA nephropathy, and evidence of calcium deposition. We believe that this case raises the clinical suspicion of sarcoidosis in patients who demonstrate findings of systemic disease without initial hypercalcemia and hilar lymphadenopathy.
